# Compound Astragalus Oral Liquid Attenuates Ventricular Remodeling in Heart Failure After Myocardial Infarction via the AMPK/TGF‐*β*/Smad Signaling Pathway

**DOI:** 10.1155/bmri/1276968

**Published:** 2026-10-18

**Authors:** Zhijun Yang, Fengfan Huang, Ji Xu, Ning Gu, Fuming Liu

**Affiliations:** ^1^ Changzhou Hospital Affiliated to Nanjing University of Chinese Medicine Changzhou Jiangsu China; ^2^ Nanjing Hospital of Chinese Medicine Affiliated to Nanjing University of Chinese Medicine Nanjing Jiangsu China njucm.edu.cn; ^3^ Jiangsu Province Hospital of Chinese Medicine Affiliated Hospital of Nanjing University of Chinese Medicine Nanjing Jiangsu China njucm.edu.cn

**Keywords:** acute myocardial infarction, AMPK, Compound Astragalus Oral Liquid, heart failure, TGF*β*

## Abstract

**Background:**

Ventricular remodeling following acute myocardial infarction (AMI) is a critical cause of heart failure (HF). Compound Astragalus Oral Liquid (CAOL) is a traditional Chinese medicine formulation with anti‐inflammatory, antioxidant, and tonifying properties. However, its effects and underlying mechanisms in post‐acute myocardial infarction heart failure (AMI‐HF) remain unclear.

**Objective:**

To evaluate the cardioprotective efficacy of CAOL in a rat model of AMI‐HF and elucidate the associated molecular mechanisms.

**Methods:**

CAOL constituents were identified by UPLC‐Q‐TOF/MS, and network pharmacology predicted potential targets. A rat model of AMI‐induced HF was established by left anterior descending (LAD) coronary ligation. Animals received CAOL at low, medium, and high doses, or perindopril. Cardiac function, histopathology, serum oxidative and inflammatory markers, and myocardial molecular expression were assessed by echocardiography, staining, ELISA, Western blotting, and RT‐qPCR.

**Results:**

CAOL treatment improved cardiac performance in a dose‐dependent manner, as evidenced by increased LVEF and improvements in LVIDd and LVIDs. Histological analyses demonstrated that CAOL reduced myocardial necrosis, inflammatory cell infiltration, and collagen deposition in a dose‐dependent fashion. CAOL also ameliorated serum markers of inflammation and oxidative stress. Mechanistically, CAOL modulated AMPK activity and inhibited TGF‐*β*1/Smad‐mediated profibrotic signaling, consistent with its antifibrotic and cytoprotective effects.

**Conclusions:**

CAOL ameliorates postinfarction ventricular remodeling and preserves cardiac function in a manner associated with AMPK activation and suppression of TGF‐*β*/Smad‐mediated fibrosis. These findings provide experimental evidence supporting CAOL as a potential therapeutic agent for AMI‐HF.

## 1. Introduction

Acute myocardial infarction (AMI) remains a leading cause of cardiovascular mortality and disability worldwide [1, 2]. Although contemporary reperfusion strategies have markedly reduced early mortality, post‐AMI ventricular remodeling continues to constitute the principal pathological substrate driving progression to chronic heart failure (HF) [3, 4]. The incidence of HF is rising annually, imposing substantial medical and socioeconomic burdens. Accumulating evidence indicates that postinfarction remodeling involves cardiomyocyte loss, inflammatory activation, compensatory hypertrophy, and stromal cell‐mediated extracellular matrix (ECM) deposition and fibrosis, ultimately resulting in chamber dilation and systolic/diastolic dysfunction [5–8]. Therapeutic strategies that limit maladaptive remodeling, suppress myocardial fibrosis, and modulate inflammation‐oxidative stress are therefore central to preventing and attenuating post‐acute myocardial infarction heart failure (AMI‐HF). Current guideline‐directed pharmacotherapy for AMI‐HF includes renin–angiotensin system inhibitors (ACE inhibitors, ARBs, and ARNIs), *β*‐blockers, SGLT2 inhibitors, and mineralocorticoid receptor antagonists [7, 9], which can delay progression, relieve symptoms, and reduce rehospitalization but are limited by adverse effects, such as hypotension, bradycardia, or renal dysfunction, that may prevent some patients from achieving target doses or tolerating treatment. Thus, there remains an unmet need for safe, multitarget interventions capable of more effectively modulating the pathophysiological processes underlying ventricular remodeling.

Traditional Chinese medicine (TCM), characterized by its multicomponent, multitarget, and multipathway pharmacological properties, has demonstrated unique therapeutic potential in managing complex diseases. Herbal formulae, in particular, have a long‐standing history and rich clinical experience in the prevention and treatment of cardiovascular disorders [10–12]. Compound Astragalus Oral Liquid (CAOL) is a standardized formulation developed from renowned Menghe School of TCM, primarily composed of *Astragalus membranaceus*, *Panax ginseng*, *Polygonatum sibiricum*, and *Pericarpium citri reticulatae*. Traditionally, it is used to tonify qi, enhance circulation, and regulate immune function. Modern pharmacological studies have shown that its main bioactive constituents—including astragalosides from *Astragalus*, ginsenosides from *P. ginseng*, and polysaccharides from *Polygonatum—*possess immunomodulatory, anti‐inflammatory, antioxidant, and cardioprotective activities [13–16]. Preliminary clinical observations have indicated that CAOL can enhance immune function and improve blood circulation in patients with coronary artery disease and atherosclerosis, with beneficial effects also observed in ischemic conditions such as myocardial or cerebral infarction. Nevertheless, its efficacy and specific molecular mechanisms in AMI‐HF have not yet been systematically elucidated, underscoring the need for in‐depth experimental investigation.

Accumulating evidence identifies the transforming growth factor‐*β* (TGF‐*β*)/Smad signaling cascade as a central regulator of fibrogenesis and ECM deposition [17, 18]. Upon ligand binding, TGF‐*β* activates receptor‐mediated phosphorylation of Smad2/3, which then complexes with Smad4 and translocates into the nucleus to induce the transcription of collagen Type I and III genes, promoting fibroblast‐to‐myofibroblast differentiation and myocardial interstitial fibrosis [19, 20]. Meanwhile, the energy sensor AMP‐activated protein kinase (AMPK) has emerged as an important modulator of cardiac metabolism and antifibrotic responses. Activation of AMPK contributes to maintaining energy homeostasis, suppressing inflammation, and attenuating profibrotic signaling through multiple downstream pathways, making it a promising target for cardioprotection [21, 22]. The potential synergistic regulation between AMPK activation and inhibition of TGF‐*β*/Smad signaling provides both theoretical and practical value for preventing ventricular remodeling. Accordingly, this study integrates ultraperformance liquid chromatography coupled with quadrupole time‐of‐flight tandem mass spectrometry (UPLC‐Q‐TOF/MS)–based component identification, network pharmacology prediction, and in vivo molecular validation to clarify the effects of CAOL on cardiac function, myocardial pathology, and inflammatory/oxidative stress responses, thereby elucidating its cardioprotective mechanisms as an antiremodeling therapy and providing experimental evidence for future active‐component screening and clinical translation.

## 2. Materials and Methods

### 2.1. Preparation of CAOL and Chemical Composition Analysis

CAOL is an oral formulation prepared from a standardized traditional herbal prescription and was supplied by the Pharmacy Department of Changzhou Hospital of Traditional Chinese Medicine (Lot No. Z04000723). The industrial batch formula comprises the following fixed dry crude herbs: *A. membranaceus* (300 g), *P. ginseng* (100 g), *P. sibiricum* (200 g), and *Pericarpium citri reticulatae* (100 g), yielding a total crude drug weight of 700 g per batch and strictly maintaining the mass ratio of 6:3:2:1. For preparation, all raw herbs were soaked in purified water for 30 min, followed by two cycles of decoction. The combined filtrates were pooled and concentrated under reduced pressure to a final constant volume of 1000 mL to obtain the bulk stock solution. The finished product was dispensed into 10‐mL single‐dose vials, with each vial corresponding to the standard single oral dose for adult humans, in accordance with the hospital′s internal preparation standard.

Chemical profiling of CAOL was performed using UPLC‐Q‐TOF/MS. Metabolite extraction from the TCM preparation was performed by mixing the medicinal solution with ethanol, followed by vortexing, sonication, overnight standing at 4°C, and centrifugation. The resulting supernatant was concentrated under nitrogen gas, reconstituted with ultrapure water, and filtered prior to analysis. LC‐MS/MS analysis was conducted on a SHIMADZU‐LC30 UHPLC system coupled to a TripleTOF 6600 mass spectrometer. Chromatographic separation was achieved using an ACQUITY UPLC HSS T3 column with a gradient elution of water and acetonitrile, both containing 0.1% formic acid. Mass spectrometry data were acquired in both positive and negative electrospray ionization modes. Raw data were processed using MS DIAL software for peak alignment, retention time correction, and peak extraction, with identification parameters set to a mass tolerance of < 0.01 Da for MS1 and < 0.02 Da for MS2, and a minimum MS2 spectrum matching score of > 70%. Statistical analysis and visualization were performed using R software.

### 2.2. Animals and Treatment

Male Sprague–Dawley rats (200–250 g) were obtained from the Animal Center of Nanjing University of Chinese Medicine. Animals were housed under specific pathogen‐free (SPF) conditions (22°C ± 2°C, relative humidity 55% ± 5%, 12‐h light/dark cycle) with ad libitum access to standard chow and water. All experimental procedures were approved by the Animal Ethics Committee of Nanjing University of Chinese Medicine (Approval No. A240110) and complied with the NIH Guide for the Care and Use of Laboratory Animals. After acclimation, rats were randomized into six groups (*n* = 12 per group): sham operation (sham), AMI‐HF model, perindopril treatment (1 mg·kg^−1^·day^−1^), and CAOL low‐dose (0.5 mL·kg^−1^·day^−1^), medium‐dose (1 mL·kg^−1^·day^−1^), and high‐dose (2 mL·kg^−1^·day^−1^) groups. Treatment commenced 24 h after surgery and was administered once daily by oral gavage for 28 consecutive days. A schematic overview of the experimental design and sample allocation is provided in Figure S2. CAOL dosing for rats was calculated using the human‐to‐rat conversion: rat dose (mg · kg^−1^) = human dose (mg · kg^−1^) × 6.2 [23]. During the 28‐day intragastric intervention, the general activity, fur condition, food intake, and body weight of all rats were recorded every 7 days. No unnatural death, lethargy, alopecia, or anorexia was observed in any CAOL treatment group.

### 2.3. Induction of AMI‐HF Model

Following an acclimatization period, a rat model of AMI‐HF was established by permanent ligation of the left anterior descending (LAD) coronary artery, as previously described with minor modifications [24, 25]. Briefly, rats were anesthetized with pentobarbital sodium (2% solution, 50 mg·kg^−1^, i.p.). After the loss of pedal reflexes, tracheal intubation was performed, and the animals were mechanically ventilated using a rodent ventilator. A left thoracotomy was performed to expose the heart, and the proximal LAD was ligated with 6‐0 silk suture. Immediate blanching of the local myocardium distal to the ligature was taken as an indicator of successful infarction. Sham‐operated animals underwent the same procedure except that the suture was passed under the LAD without ligation. The chest was closed in layers, endotracheal tube removed upon recovery of spontaneous respiration, and animals returned to standard housing.

### 2.4. Echocardiography Evaluation

Transthoracic echocardiography was performed before surgery and at the end of the treatment period using an Esaote MyLab small‐animal ultrasound system (*n* = 8 per group). Rats were lightly anesthetized with isoflurane, and parasternal long‐axis M‐mode images were obtained at the level of the papillary muscles. Representative M‐mode images with labeled interventricular septal thickness (IVS), left ventricular internal diameter (LVID), and left ventricular posterior wall thickness (LVPW) are provided in Figure S3. Left ventricular end‐systolic internal diameter (LVIDs), left ventricular end‐diastolic internal diameter (LVIDd), and left ventricular ejection fraction (LVEF) were measured from M‐mode tracings. LVEF was calculated using the Teichholz formula: LVEF (*%*) = (EDV − ESV)/EDV × 100*%*.

### 2.5. Sample Collection

At the end of the experiment, animals were euthanized under anesthesia and blood was collected from the abdominal aorta. After standing and centrifugation, serum was aliquoted and stored at −80°C until assay. Hearts were rapidly excised; the left ventricle was dissected, and portions were fixed in 4% paraformaldehyde for histology, whereas remaining tissue samples were snap‐frozen in liquid nitrogen and stored at −80°C for molecular analyses.

### 2.6. Serum Biochemical Measurement

Serum concentrations of interleukin‐6 (IL‐6), interleukin‐10 (IL‐10), tumor necrosis factor‐*α* (TNF‐*α*), and interferon‐*γ* (IFN‐*γ*), together with superoxide dismutase (SOD) activity and malondialdehyde (MDA) levels, were quantified using commercial ELISA kits (Nanjing Jiancheng, China) according to the manufacturers′ instructions. To evaluate the in vivo safety of CAOL, serum alanine aminotransferase (ALT), aspartate aminotransferase (AST), creatinine (CREA), and urea nitrogen (UREA) levels were measured using an automatic biochemical analyzer (Hitachi 7180, Japan) with commercial kits (Nanjing Jiancheng, China).

### 2.7. Hematoxylin and Eosin (H&E) and Masson Trichrome Staining

Following fixation and dehydration, cardiac tissues were embedded in paraffin and sectioned at a thickness of 4–5 *μ*m. The sections were subsequently subjected to H&E and Masson trichrome staining according to standard protocols (*n* = 5 biological replicates per group). Histological images were acquired using a Leica DM2000 light microscope (Wetzlar, Germany). The collagen volume fraction was quantified using ImageJ software by measuring the blue‐stained fibrotic area as a percentage of the total tissue area in each section; five randomly selected fields per animal were analyzed and averaged.

### 2.8. TUNEL Staining

After antigen retrieval with proteinase K, tissue sections were processed using a TUNEL assay kit (Roche, Germany) following the manufacturer′s instructions. TUNEL reaction mixture was added to each section and incubated at 37°C for 60 min in the dark. Nuclei were counterstained with DAPI, and images were captured using a NIKON ECLIPSE microscope (Tokyo, Japan). The percentage of TUNEL‐positive cardiomyocytes was calculated by counting TUNEL‐positive nuclei and DAPI‐stained total nuclei in five randomly selected high‐power fields per section, with three sections per animal; cardiomyocytes were identified by their typical elongated morphology and central nuclei. The percentage was expressed as (TUNEL − positive nuclei/total nuclei) × 100*%*.

### 2.9. Real‐Time Quantitative PCR (RT‐qPCR)

Total RNA was isolated from frozen cardiac tissues using TRIzol reagent. cDNA synthesis and RT‐qPCR reactions were performed with a reverse transcription kit and SYBR Green Master Mix (YEASEN, China), following the corresponding manuals. The mRNA expression levels of AMPK, TGF‐*β*1, Smad2, and Smad3 were normalized to GAPDH. All primer sequences are listed in Table S1.

### 2.10. Western Blot (WB)

Cardiac tissues were lysed on ice using RIPA buffer (Beyotime, China) supplemented with protease inhibitors, and total protein concentrations were measured using a BCA assay. Equal amounts of protein were separated via SDS‐PAGE and transferred onto PVDF membranes (Millipore, United States). After blocking with nonfat milk at room temperature, membranes were incubated overnight at 4°C with primary antibodies against AMPK (CST, United States), p‐AMPK (CST, United States), TGF‐*β*1 (Proteintech, China), Smad2 (Proteintech, China), Smad3 (Proteintech, China), p‐Smad2 (CST, United States), p‐Smad3 (CST, United States), and GAPDH (Proteintech, China). The following day, membranes were incubated with HRP‐conjugated secondary antibodies (Proteintech, China) for 1 h at room temperature. Protein bands were visualized using an ECL chemiluminescent detection system, and band intensities were quantified using ImageJ software.

### 2.11. Network Pharmacology

Potential active ingredients and their targets were identified and screened based on UPLC‐Q‐TOF/MS results and through the Traditional Chinese Medicine Systems Pharmacology Database and Analysis Platform (TCMSP, https://old.tcmsp-e.com/tcmsp.php). The screening criteria were set as oral bioavailability (OB) ≥ 30% and drug‐likeness (DL) ≥ 0.18. The TCMSP platform was used to predict the potential protein targets of these compounds, which were then standardized to gene symbols using the UniProt database (https://www.uniprot.org). Additionally, disease‐related targets were retrieved using the keyword “post‐acute myocardial infarction heart failure” from the GeneCards database (https://www.genecards.org).

The common targets between CAOL and the disease were identified using a Venn diagram. A protein–protein interaction (PPI) network was constructed using the STRING database (https://cn.string-db.org) with a confidence score > 0.9, and visualized with Cytoscape software (Version 3.10.1). Topological analysis was performed using the CytoNCA plugin to identify core targets based on centrality parameters such as degree, betweenness, and closeness. Gene ontology (GO) and Kyoto Encyclopedia of Genes and Genomes (KEGG) pathway enrichment analyses for the intersecting targets were conducted using the Metascape platform (https://metascape.org).

### 2.12. Statistical Analysis

Data are expressed as mean ± standard deviation (SD). Statistical analysis was performed using GraphPad Prism software (Version 8.0). One‐way analysis of variance (ANOVA) was used for comparisons among multiple groups, and post hoc testing was performed using Tukey′s method. A *p* value of < 0.05 was considered statistically significant.

## 3. Results

### 3.1. Component Analysis of the CAOL

Using UPLC‐Q‐TOF/MS, a systematic compositional analysis of CAOL was performed. A total of 319 compounds were identified in the positive ion mode and 128 compounds in the negative ion mode. The base peak chromatograms obtained in both modes clearly revealed the chemical complexity of CAOL (Figure 1A,B). Based on relative peak area intensities, the top 30 major components are listed in Table 1. These compounds primarily belong to flavonoids, isoflavones, ginsenosides, and triterpenoids (Figure S1), several of which have been reported to exhibit anti‐inflammatory, antioxidant, antifibrotic, and cardioprotective activities [14, 26, 27]. This comprehensive chemical profiling provides valuable evidence for understanding the phytochemical characteristics of CAOL and establishes a molecular foundation for subsequent network pharmacology analyses and mechanistic studies, given its multitarget and synergistic pharmacological properties.

**Figure 1:**
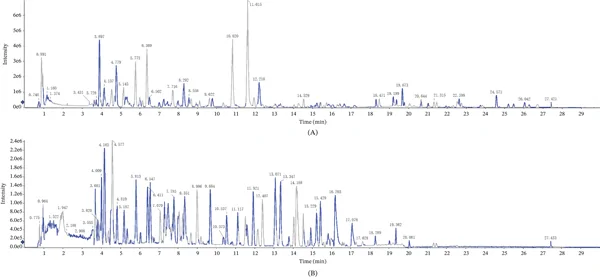
UPLC‐Q‐TOF/MS analysis of CAOL. (A) Base peak chromatogram, positive ion mode. (B) Base peak chromatogram, negative ion mode.

**Table 1:** Top 30 primary chemical components in CAOL.

Number	RT	Ion mode	Identification	m/z	Formula	Class
1	11.612	[M + H]+	3,5,7,8,3 ^′^,4 ^′^,5 ^′^‐Heptamethoxyflavone	433.1499	C_22_H_24_O_9_	Flavonoids
2	10.82	[M + H]+	Nobiletin	403.13977	C_21_H_22_O_8_	Flavonoids
3	13.341	[M−H]^−^	Ginsenoside Rg3	783.4903	C_42_H_72_O_13_	Prenol lipids
4	4.78	[M + H]+	Ononin	431.13501	C_22_H_22_O_9_	Isoflavonoids
5	4.14	[M + H]+	Hesperidin	611.19635	C_28_H_34_O_15_	Flavonoids
6	3.901	[M + H]+	Calycosin 7‐O‐glucoside	447.12985	C_22_H_22_O_10_	Isoflavonoids
7	0.89	[M + H]+	Arginine	175.11858	C_6_H_14_N_4_O_2_	Carboxylic acids and derivatives
8	7.704	[M + HCOO]^−^	Ginsenoside Rb2	1123.59595	C_53_H_90_O_22_	Prenol lipids
9	8.295	[M + H]+	Formononetin	269.08176	C_16_H_12_O_4_	Isoflavonoids
10	5.777	[M + H]+	Calycosin	285.0755	C_16_H_12_O_5_	Isoflavonoids
11	15.23	[M + FA−H]^−^	Ginsenoside Rg5	811.48663	C_42_H_70_O_12_	Prenol lipids
12	4.576	[M + FA−H]^−^	Ginsenoside Rg1	845.49286	C_42_H_72_O_14_	Prenol lipids
13	12.217	[M + H]+	Tangeretin	373.13046	C_20_H_20_O_7_	Flavonoids
14	7.245	[M + Na]+	Ginsenoside Rb1	1131.59436	C_54_H_92_O_23_	Prenol lipids
15	6.544	[M−H]^−^	Ginsenoside Rf	799.48566	C_42_H_72_O_14_	Glycerolipids
16	1.203	[M + Na]+	Sucrose	365.10489	C_12_H_22_O_11_	Organooxygen compounds
17	4.532	[M + H]+	Glochidone putative	423.36185	C_30_H_46_O	Prenol lipids
18	5.143	[M + H]+	3‐Hydroxy‐9,10‐Dimethoxypterocarpan	301.10703	C_17_H_16_O_5_	Isoflavonoids
19	7.726	[M + H−H2O]+	Oleanolic acid	439.35712	C_30_H_48_O_3_	Prenol lipids
20	5.809	[M−H]^−^	Biochanin A	283.06143	C_16_H_12_O_5_	Isoflavonoids
21	8.981	[M + HCOO]^−^	Ginsenoside B2	991.55157	C_48_H_82_O_18_	Prenol lipids
22	0.913	[M + Na]+	Theophylline	203.05217	C_7_H_8_N_4_O_2_	Imidazopyrimidines
23	7.776	[M−H]^−^	Ginsenoside Ro	955.49304	C_48_H_76_O_19_	Prenol lipids
24	4.011	[M−H]^−^	Narirutin	579.17212	C_27_H_32_O_14_	Flavonoids
25	1.091	[M−H]^−^	Citric acid	191.01932	C_6_H_8_O_7_	Carboxylic acids and derivatives
26	7.249	[M + H−H2O]+	Betulin	425.37875	C_30_H_50_O_2_	Prenol lipids
27	19.675	[M + H]+	Oleamide	282.28018	C_18_H_35_NO	Fatty Acyls
28	11.616	[M + Na]+	3,3 ^′^,4 ^′^,5,6,7,8‐Heptamethoxyflavone	455.13162	C_22_H_24_O_9_	Flavonoids
29	7.995	[M + FA−H]^−^	Ginsenoside F1	683.43774	C_36_H_62_O_9_	Prenol lipids
30	9.627	[M + H]+	Sinensetin	373.12723	C_20_H_20_O_7_	Flavonoids

### 3.2. CAOL Improves Cardiac Function and Attenuates Ventricular Remodeling in AMIHF Rats

Rats subjected to LAD ligation exhibited marked cardiac dysfunction and structural damage characteristic of AMI‐induced HF. Echocardiographic analysis showed that compared with the sham group, the model group displayed a significant reduction in LVEF and marked increases in LVIDd and LVIDs (*p* < 0.05), indicating ventricular dilation and impaired systolic function (Figure 2A–D). After 28 days of CAOL administration, cardiac function improved substantially. LVEF increased, and LVIDd and LVIDs decreased in a dose‐dependent manner across all treatment groups, with the high‐dose CAOL group showing effects comparable with those of the positive control drug, perindopril.

**Figure 2:**
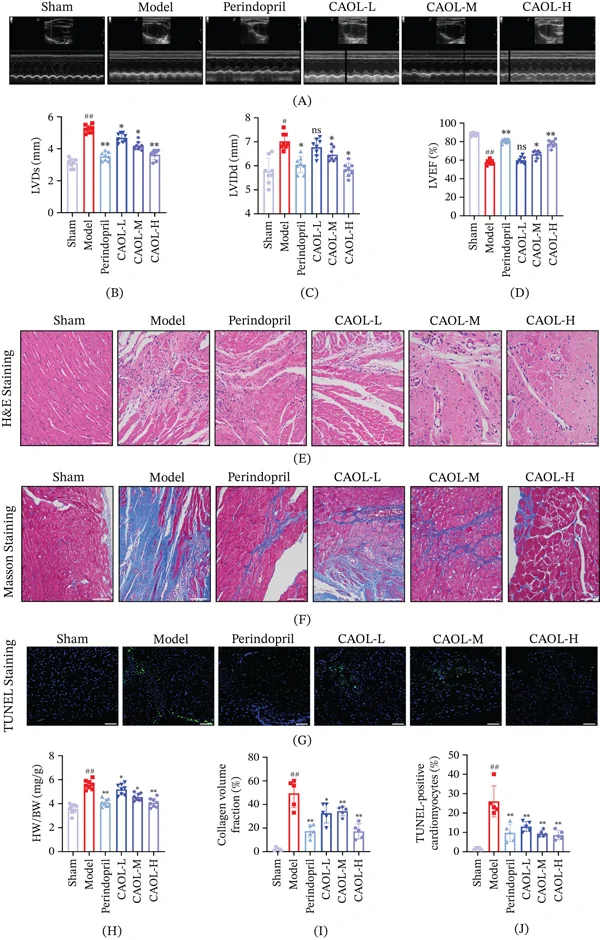
CAOL improves cardiac function and attenuates pathological remodeling and myocardial injury in AMI‐HF rats. (A) Representative M‐mode echocardiographic images. (B–D). Quantification results of LVIDs, LVIDd, and LVEF (*n* = 8). (E) Representative images of H&E staining. Scale bar = 200 *μ*m. (F) Representative images of Masson trichrome staining, blue stain represents fibrous tissue and collagen deposition. Scale bar = 200 *μ*m. (G) Representative images of TUNEL staining. Scale bar = 200 *μ*m. (H) The heart weight/body weight ratio of rats (*n* = 8). (I) Collagen volume fraction of Masson trichrome staining (*n* = 5). (J) Quantification of TUNEL‐positive staining (*n* = 5). Data were shown as mean ± SD, ^#^ *p* < 0.05, ^##^ *p* < 0.01 compared with the sham group. ∗*p* < 0.05, ∗∗*p* < 0.01 compared with the model group, ns means nonsignificant.

Histopathological results were consistent with echocardiographic findings. H&E staining revealed disorganized myocardial fibers, necrotic areas, and inflammatory cell infiltration in the model group, whereas CAOL‐treated hearts exhibited relatively preserved myocardial architecture and markedly reduced inflammation (Figure 2E). Masson trichrome staining further demonstrated extensive collagen deposition and prominent fibrotic areas in the model group, whereas CAOL treatment dose‐dependently reduced pathological collagen accumulation (Figures 2F,I and S4A). Additionally, an increased heart weight to body weight ratio (HW/BW) served as an indicator of cardiac hypertrophy. This ratio was significantly elevated in the model group compared with the sham group, whereas CAOL treatment dose‐dependently lowered HW/BW (Figure 2H). These macroscopic findings were corroborated by histological examination of H&E‐stained sections at higher magnification (Figure S4B), which revealed marked cardiomyocyte hypertrophy in the model group and dose‐dependent morphological improvement following CAOL treatment, consistent with the changes observed in HW/BW ratio. TUNEL staining showed a marked increase in cardiomyocyte apoptosis in the model group, which was attenuated by CAOL administration (Figure 2G,J). Collectively, these results indicate that CAOL effectively ameliorates post‐AMI cardiac dysfunction and attenuates ventricular remodeling.

### 3.3. CAOL Suppresses Serum Inflammation and Oxidative Stress in AMI‐HF Rats

To further clarify the systemic regulatory effects of CAOL in AMI‐HF rats, serum inflammatory cytokines and oxidative stress markers were assessed. Compared with the sham group, the model group exhibited a pronounced proinflammatory shift, characterized by significantly elevated levels of IL‐6, TNF‐*α*, and IFN‐*γ* (*p* < 0.05), along with a marked decline in the anti‐inflammatory cytokine IL‐10 (Figure 3A–D). In addition, oxidative stress was aggravated, as reflected by reduced SOD activity and increased MDA levels (Figure 3E,F). These biochemical alterations are consistent with the persistent systemic inflammation and oxidative injury typically observed after myocardial infarction. Importantly, CAOL treatment effectively reversed these pathological changes. IL‐6, TNF‐*α*, and IFN‐*γ* levels were significantly reduced, whereas IL‐10 was upregulated, indicating a restoration of inflammatory balance. SOD activity was significantly restored, and MDA levels decreased (*p* < 0.05), demonstrating clear anti‐inflammatory and antioxidant effects. The medium‐ and high‐dose CAOL groups showed improvements comparable with or exceeding those achieved with perindopril. Collectively, these findings suggest that CAOL mitigates systemic inflammatory activation and oxidative stress, thereby contributing to a more favorable internal milieu for myocardial repair.

**Figure 3:**
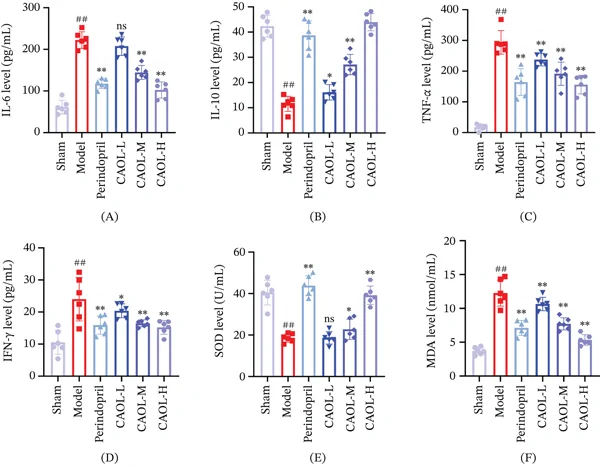
Systemic effects of CAOL on circulating biomarkers of inflammation and oxidative stress. (A) IL‐6, (B) IL‐10, (C) TNF‐*α*, (D) IFN‐*γ*, (E) SOD, and (F) MDA levels with administration of CAOL in serum were analyzed. Data were shown as mean ± SD (*n* = 6), ^##^ *p* < 0.01 compared with the sham group. ∗*p* < 0.05, ∗∗*p* < 0.01 compared with the model group, ns means nonsignificant.

Furthermore, to assess the in vivo safety profile of CAOL, serum biochemical parameters reflecting liver and kidney function were measured at the end of the 28‐day treatment period. As shown in Figure S5, serum ALT, AST, CREA, and UREA levels in the model group were slightly elevated compared with the sham group, which is consistent with the systemic stress response following myocardial infarction. Notably, CAOL treatment at all three doses did not significantly increase any of these parameters relative to the model group. These findings demonstrate that CAOL administration at the tested doses does not induce detectable hepatic or renal injury, supporting the favorable safety profile of this herbal formulation.

### 3.4. Network Pharmacology Indicates Potential Signaling Pathways Underlying CAOL Activity

Based on the phytochemical components identified by UPLC‐Q‐TOF/MS, a total of 255 putative CAOL targets were screened from the TCMSP and UniProt databases. A total of 3949 disease‐related targets associated with AMI‐HF were obtained from the GeneCards database. Intersection analysis revealed 191 overlapping targets between CAOL and the disease (Figure 4A), suggesting that CAOL may regulate multiple pathological processes relevant to AMI‐HF. A PPI network was subsequently constructed via STRING and visualized in Cytoscape, comprising 301 nodes (including 4 herbal nodes and 42 active compound nodes) and 792 edges (Figure 4B). Topological analysis based on degree, betweenness, and closeness centrality identified 20 core targets (Table 2), including TGF‐*β*1, TNF, AKT1, TP53, IL‐6, and IL‐10. These targets are known to play essential roles in inflammation, fibrosis, apoptosis, and energy metabolism, implying that CAOL exerts its pharmacological effects through multitarget modulation.

**Figure 4:**
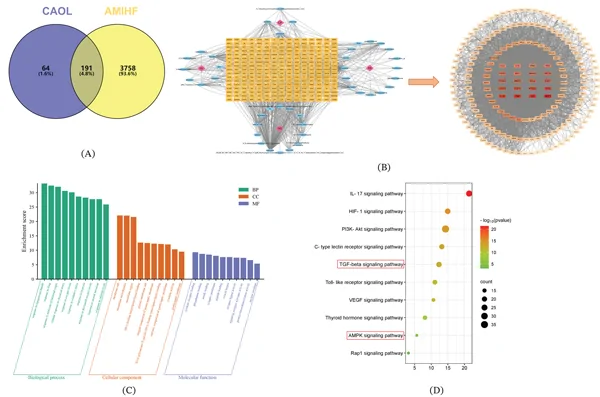
Network pharmacology analysis reveals potential targets and mechanisms of CAOL against AMI‐HF. (A) Venn diagram of compound targets shared by CAOL and AMIHF. (B) The PPI network of intersection targets and topological screening process of the active ingredient−target−disease network diagram. (C) GO enrichment analysis of the anti‐AMIHF targets of CAOL. (D) KEGG enrichment analysis for anti‐AMIHF targets of CAOL.

**Table 2:** Potential key targets for the effects of CAOL.

	Degree	BC	CC
AKT1	135	1514.635027	0.004166667
IL6	131	1213.702373	0.004098361
TNF	131	1304.413461	0.004098361
IL1B	123	920.2361121	0.003968254
TP53	120	788.3370058	0.003891051
PTGS2	116	1552.004445	0.003875969
EGFR	113	1023.964527	0.003802281
CASP3	112	481.3752703	0.003773585
ESR1	111	1150.543168	0.003773585
BCL2	109	420.0943103	0.003745318
MMP9	108	408.9622157	0.003690037
HIF1A	107	415.2880553	0.003690037
PPARG	107	659.5177308	0.003717472
MAPK3	102	391.361716	0.003610108
MYC	99	399.6008114	0.003584229
TGFB1	99	255.6376045	0.003584229
FOS	97	765.9352577	0.003610108
IFNG	94	220.1291079	0.003521127
IL10	93	281.5031342	0.003521127
CCL2	92	205.3707229	0.003472222

GO enrichment analysis (Figure 4C) revealed that the intersecting targets were primarily involved in oxidative stress responses, apoptotic regulation, inflammatory pathways, and metabolic processes. KEGG pathway analysis (Figure 4D) indicated significant enrichment in pathways such as TGF‐*β*, AMPK, HIF‐1, and IL‐17 signaling. These results collectively suggest that CAOL may exert anti‐inflammatory, antioxidant, and antifibrotic functions through coordinated regulation of the AMPK and TGF‐*β*/Smad pathways, linking to the pathophysiological features observed in AMI‐HF.

### 3.5. CAOL Modulates the AMPK and TGF‐*β*/Smad Signaling Pathways in Myocardial Tissue

To validate the predictions from network pharmacology, we assessed the expression of key targets within the relevant pathways in myocardial tissues. RT‐qPCR results demonstrated that AMPK mRNA levels were significantly decreased in the model group compared with the sham group, whereas the mRNA expression of TGF‐*β*1, Smad2, and Smad3 was markedly increased (Figure 5A–D). CAOL treatment, particularly at medium and high doses, effectively restored AMPK expression while suppressing TGF‐*β*1, Smad2, and Smad3 transcription, indicating a corrective effect on transcriptional dysregulation induced by AMI‐HF. Consistent with the mRNA findings, WB analysis revealed a substantial reduction in AMPK protein expression and a decreased p‐AMPK/AMPK ratio in the model group (Figure 5E–G), alongside elevated levels of TGF‐*β*1 and phosphorylated Smad2/3 (Figure 5H–L). CAOL administration dose‐dependently enhanced AMPK phosphorylation and reduced TGF‐*β*1 and p‐Smad2/3 protein levels. Taken together, these results suggest that CAOL treatment is associated with activation of AMPK signaling and inhibition of the aberrant activation of the TGF‐*β*/Smad pathway in the myocardium, correlating with the attenuation of myocardial fibrosis and ventricular remodeling in AMI‐HF rats. These molecular changes align with the improvements in cardiac structure, function, and inflammatory‐oxidative status observed in earlier results, supporting a coherent mechanistic framework for CAOL′s cardioprotective effects.

**Figure 5:**
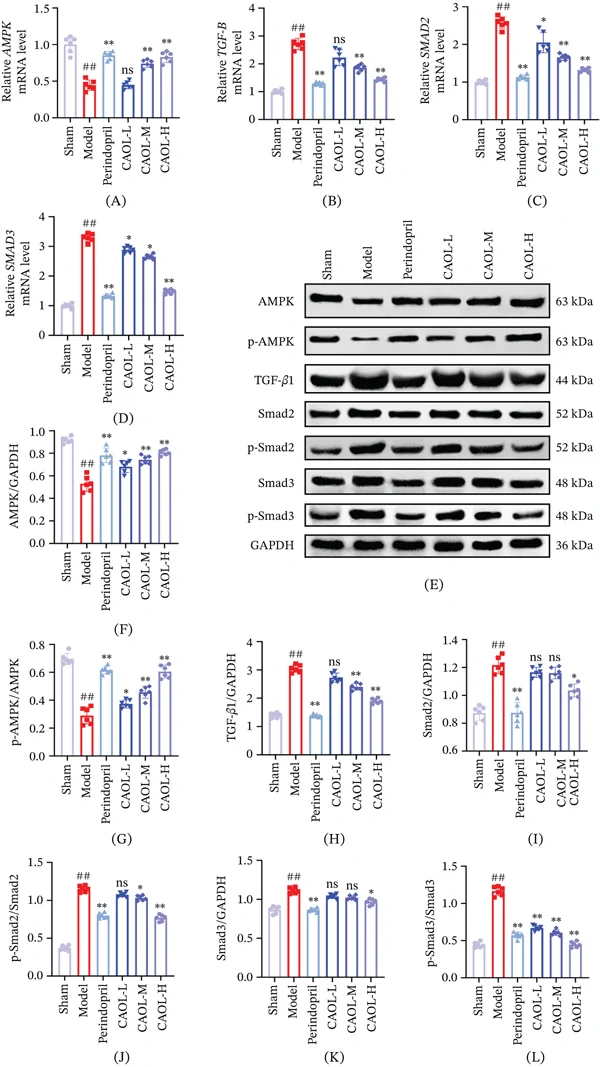
CAOL enhances AMPK activity and suppresses the TGF‐*β*/Smad pathway in myocardial tissue. (A–D) The mRNA levels of *AMPK*, *TGF-β*, *SMAD2*, and *SMAD3* were detected, respectively, by RT‐qPCR (*n* = 6). (E–L) WB analysis and quantification of the protein levels of AMPK, p‐AMPK, TGF‐*β*1, Smad2, p‐Smad2, Smad3, and p‐Smad3 (*n* = 6). Data were shown as mean ± SD, ^##^ *p* < 0.01 compared with the sham group. ∗*p* < 0.05, ∗∗*p* < 0.01 compared with the model group, ns means nonsignificant.

## 4. Discussion

This study provides a comprehensive evaluation of the cardioprotective effects of CAOL in a rat model of AMI‐HF. Through the integrated analysis of chemical profiling, network pharmacology, and molecular validation, we demonstrate that CAOL exerts robust therapeutic benefits via a multicomponent, multitarget, and multipathway mechanism. Specifically, CAOL ameliorates cardiac dysfunction, attenuates myocardial fibrosis, and restores systemic inflammatory and oxidative balance. Importantly, our findings highlight the concurrent activation of the AMPK pathway and suppression of the TGF‐*β*/Smad axis as key molecular events associated with the beneficial effects of CAOL, two pathways that play intertwined roles in postinfarction remodeling and fibrotic progression.

Following myocardial infarction, chronic inflammation, metabolic disruption, and progressive fibrosis constitute a pathological triad that drives adverse ventricular remodeling and functional deterioration. In this context, CAOL produced marked improvements in cardiac function—evidenced by the elevation of LVEF and reductions in LVIDd and LVIDs—which were closely associated with reduced cardiomyocyte necrosis, diminished inflammatory infiltration, and significantly decreased collagen deposition (Figure 2). The concordance between functional recovery and histological repair suggests that CAOL does more than alleviate symptoms; it intervenes at structural and cellular levels to reverse maladaptive remodeling. Notably, its therapeutic efficacy was comparable with that of the ACE inhibitor perindopril, underscoring the potential of CAOL as a novel multitarget adjunctive therapy for AMI‐HF. Although perindopril treatment was associated with increased AMPK phosphorylation in our study, this effect likely reflects an indirect consequence of reduced oxidative and inflammatory stress—a well‐recognized secondary effect of ACE inhibitors—rather than direct AMPK activation. CAOL also exerted broad immunomodulatory and antioxidant effects, as indicated by the reduction of proinflammatory cytokines (IL‐6, TNF‐*α*, and IFN‐*γ*), elevation of the anti‐inflammatory cytokine IL‐10, restoration of SOD activity, and decreased MDA levels (Figure 3). Although systemic inflammation typically peaks within the first week after myocardial infarction, accumulating evidence indicates that a sustained low‐grade inflammatory state persists into the subacute and chronic phases and is closely associated with progressive ventricular remodeling and adverse outcomes. Because inflammation and oxidative stress amplify each other in a feed‐forward cycle of myocardial injury, their simultaneous improvement suggests that CAOL disrupts this pathological reinforcement, thereby creating a biochemical environment conducive to myocardial repair. In summary, CAOL facilitates a holistic arrest of the postinfarction pathological cascade by reinstating homeostasis across metabolism, inflammation, and fibrosis. Such coordinated regulation across multiple pathological dimensions exemplifies the systemic therapeutic advantages of multicomponent TCM herbal formulations.

A major mechanistic insight from this work lies in the dual modulation of the AMPK and TGF‐*β*/Smad pathways by CAOL. AMPK is a pivotal regulator of myocardial energy homeostasis and is typically suppressed in ischemic injury and HF [22, 28, 29]. Ischemia and reperfusion injury induce mitochondrial dysfunction, ATP depletion, and excessive production of reactive oxygen species (ROS), which collectively activate multiple profibrotic signaling pathways. In this study, CAOL significantly increased p‐AMPK/AMPK levels, suggesting enhanced energy stress sensing and metabolic adaptation. AMPK activation confers cardioprotection by inhibiting NF‐*κ*B–mediated inflammation, promoting autophagic clearance of damaged organelles, and suppressing mTOR‐dependent signaling [30–32]. Moreover, accumulating evidence indicates that AMPK could directly interfere with Smad3 phosphorylation, thereby antagonizing TGF‐*β*/Smad‐driven fibrosis and establishing a mechanistic bridge between metabolic regulation and ECM homeostasiss [33, 34]. An innovative finding of our study is the concurrent modulation of AMPK activity and suppression of the TGF‐*β*/Smad pathway following CAOL treatment. Excessive TGF‐*β*1 activation is a hallmark driver of myocardial fibrosis. Upon ligand binding, TGF‐*β*1 stimulates phosphorylation of Smad2/3, which form transcriptional complexes with Smad4 to promote the expression of Collagen I, Collagen III, and other ECM proteins [17–20]. Our results demonstrated that CAOL downregulated TGF‐*β*1 and p‐Smad2/3 expression (Figure 5), effectively inhibiting the core cascade responsible for fibrotic progression. The reciprocal relationship—AMPK activation and TGF‐*β*/Smad inhibition—constitutes a coherent regulatory network that explains the observed antifibrotic and remodeling‐modulatory effects of CAOL. This network‐level coordination reflects the pharmacological essence of traditional multiherb formulations.

The observation that significant suppression of TGF‐*β*1, Smad2, and Smad3 was achieved predominantly at the high dose suggests a threshold effect for pathway inhibition, likely attributable to the limited OB of certain key active constituents, particularly astragaloside IV, which may restrict myocardial drug concentrations at lower doses. This pharmacokinetic consideration, together with potential synergistic interactions among multiple components such as ginsenosides and flavonoids, likely accounts for the requirement of higher doses to achieve effective pathway modulation. These findings underscore the importance of appropriate dosing to achieve therapeutic efficacy and provide practical guidance for clinical translation, suggesting that dose titration or formulation optimization may be necessary to attain meaningful antifibrotic effects. Among the bioactive constituents identified in CAOL (Table 1), astragaloside IV—a principal saponin from *A. membranaceus*—has been demonstrated to activate AMPK and attenuate cardiac fibrosis in ischemic heart disease models through the suppression of TGF‐*β*1 and Smad2/3 phosphorylation. Similarly, ginsenosides Rg1 and Rb1 from *P. ginseng* have been reported to exert cardioprotective effects by modulating AMPK activity and reducing oxidative stress. Furthermore, flavonoids such as calycosin and formononetin, which are abundant in CAOL, possess anti‐inflammatory and antifibrotic properties that may contribute synergistically to the overall efficacy. These findings suggest that the observed beneficial effects of CAOL are likely attributable to the collective and synergistic actions of multiple active compounds rather than a single component, which is characteristic of multiherbal TCM formulations.

Although this study elucidates a key mechanism by which CAOL ameliorates ventricular remodeling, several limitations should be considered. First, the causal relationship between AMPK activation and TGF‐*β*1 inhibition requires further clarification using pharmacological antagonists or genetic manipulation. Nevertheless, it is well established that AMPK activation suppresses TGF‐*β*/Smad signaling through multiple mechanisms, including inhibition of R‐Smad phosphorylation, suppression of transcriptional coactivator p300 activity, and upregulation of inhibitory Smad7 expression. Indeed, loss‐of‐function studies have demonstrated that AMPK inhibition or ablation greatly enhances TGF‐*β*–stimulated Smad2/3 phosphorylation, and AMPK agonists have been shown to attenuate cardiac fibrosis through suppression of TGF‐*β*1 and Smad2/3 activation. The concurrent AMPK activation and TGF‐*β*/Smad suppression observed in our study are therefore consistent with this established regulatory axis, providing a strong mechanistic rationale for CAOL′s antifibrotic effects. Second, given the multicomponent nature of CAOL, the definitive active compounds responsible for its observed efficacy require further isolation and identification. Finally, long‐term efficacy and safety of CAOL should be evaluated in chronic models and clinical studies. Nonetheless, the integrated framework established here—combining UPLC‐Q‐TOF/MS metabolite profiling, network pharmacology, and experimental validation—offers a systematic paradigm for dissecting the mechanisms of complex herbal formulations and highlights the translational potential of CAOL for preventing remodeling in AMI‐HF.

In summary, CAOL confers comprehensive protection against post‐MI cardiac dysfunction, with these effects occurring in conjunction with AMPK signaling activation, TGF‐*β*/Smad pathway inhibition, and rebalancing of inflammatory and oxidative states. This multitarget, systems‐level mode of action exemplifies the advantages of traditional herbal formulations in addressing multifactorial diseases and provides a mechanistic foundation for developing CAOL as a promising therapeutic candidate for AMI‐HF.

## 5. Conclusion

In this study, we employed an integrated experimental approach to demonstrate that CAOL significantly improves cardiac function, attenuates myocardial necrosis and fibrosis, and mitigates inflammatory and oxidative stress responses in a rat model of postinfarction HF. Mechanistically, these protective effects are closely associated with the activation of AMPK signaling and suppression of TGF‐*β*/Smad‐mediated fibrotic pathways. By coordinating multiple targets and pathways, CAOL effectively inhibits ventricular remodeling and preserves myocardial integrity. These findings provide mechanistic and pharmacological evidence supporting the use of CAOL in the management of postmyocardial infarction HF and highlight the unique therapeutic potential of traditional multicomponent herbal formulations in addressing complex cardiovascular diseases.

## Author Contributions

**Zhijun Yang:** investigation, methodology, data curation, writing – original draft. **Fengfan Huang:** investigation, data curation. **Ji Xu:** methodology, software. **Ning Gu:** data curation. **Fuming Liu:** conceptualization, funding acquisition, project administration, writing – review and editing.

## Funding

This study was supported by the Young Talent Program of Changzhou Municipal Health Commission (No. QN202237) and the Natural Science Foundation of Nanjing University of Chinese Medicine (No. XZR2024140).

## Disclosure

The authors declare that all data were generated in‐house, that no paper mill was used, and that no AI tool has been used for the generation of text or figures.

## Ethics Statement

All experimental procedures were performed in accordance with the National Institute of Health Guide for the Care and Use of Laboratory Animals, and the experimental plan was approved by the Animal Research Committee of Nanjing University of Chinese Medicine (Approval No. A240110).

## Conflicts of Interest

The authors declare no conflicts of interest.

## Supporting information


**Supporting Information** Additional supporting information can be found online in the Supporting Information section. Table S1: Primers for quantitative RT‐PCR analysis. Table S2: Catalog numbers of kits and antibodies used in this study. Figure S1: Classification diagram of compound constituents in CAOL identified by UPLC‐Q‐TOF/MS. Figure S2: Schematic diagram of the experimental design. Figure S3: Representative M‐mode echocardiographic images with anatomical labeling. Figure S4: Representative whole‐heart Masson trichrome staining images and H&E‐stained images of cardiomyocyte morphology. Figure S5: Serum biochemical parameters of liver and kidney function in each group.

## Data Availability

The data that support the findings of this study are available from the corresponding author upon reasonable request.
