# Supplementary material for: Compound Astragalus Oral Liquid Attenuates Ventricular Remodeling in Heart Failure After Myocardial Infarction via the AMPK/TGF‐β/Smad Signaling Pathway
Source: Biomed Res Int. 2026 Oct 18;2026:1276968. doi: 10.1155/bmri/1276968 (PMC13677130; doi:10.1155/bmri/1276968)
Supplement: Supplementary file 1 [file BMRI-2026-1276968-s001.docx]

**Compound Astragalus Oral Liquid attenuates ventricular remodeling in heart failure after myocardial infarction via the AMPK / TGF-β / Smad signaling pathway**

Zhijun Yang¹, Fengfan Huang¹, Ji Xu¹, Ning Gu², Fuming Liu³*

1. Changzhou Hospital Affiliated to Nanjing University of Chinese Medicine, Changzhou, Jiangsu, 213003, China.

2. Nanjing Hospital of Chinese Medicine affiliated to Nanjing University of Chinese Medicine, Nanjing, Jiangsu, 210001, China.

3. Jiangsu Province Hospital of Chinese Medicine, Affiliated Hospital of Nanjing University of Chinese Medicine, Nanjing, Jiangsu, 210001, China.

Correspondence to:

Fuming Liu*: fsyy00652@njucm.edu.cn

Supplementary Table 1 Primers for quantitative RT-PCR analysis

| Gene | Primer sequence (5'-3') | |
| --- | --- | --- |
|  | Forward | Reverse |
| AMPK | AATTCGCAGGGAGATTCAGA | ACAGCTCTCCTCCAGAAACG |
| TGF-B1 | CTGCTGACCCCCACTGATAC | AGCCCTGTATTCCGTCTCCT |
| SMAD2 | CCGCCCGAAGGGTAGAT | TGAATGGCAAGATGGACGACA |
| SMAD3 | CAGGAGGAGAAGTGGTGCGA | TCCAGTGACCTGGGGATGGTAA |
| GAPDH | TCGTGGAGTCTACTGGCGTCTT | CATTGCTGACAATCTTGAGGGAG |

For each gene, the table provides the forward and reverse primer sequences in the 5′ to 3′ direction. These primers were employed for quantitative real-time PCR (qPCR) analysis of target gene expression in rat hearts, which is described in *2.9. Real-Time Quantitative PCR* of the main text.

Supplementary Table 2 Catalog numbers of kits and antibodies used in this study

| **Reagents/Antibodies** | **Brand** | **Catalog** |
| --- | --- | --- |
| IL-6 | Nanjing Jiancheng | H007-1-2 |
| IL-10 | Nanjing Jiancheng | H009-1-2 |
| TNF-α | Nanjing Jiancheng | H052-1-2 |
| IFN-γ | Nanjing Jiancheng | H025-1-2 |
| SOD | Nanjing Jiancheng | A001-3 |
| MDA | Nanjing Jiancheng | A003-1-2 |
| ALT | Nanjing Jiancheng | C009-2-1 |
| AST | Nanjing Jiancheng | C010-2-1 |
| CREA | Nanjing Jiancheng | C011-2-1 |
| UREA | Nanjing Jiancheng | C013-1-1 |
| AMPKα | Cell Signaling Technology | 2532 |
| p-AMPKα | Cell Signaling Technology | 2535 |
| TGF-β1 | Proteintech | 21898-1-AP |
| Smad2 | Proteintech | 12570-1-AP |
| Smad3 | Proteintech | 25494-1-AP |
| p-Smad2 | Cell Signaling Technology | 3108 |
| p-Smad3 | Cell Signaling Technology | 9520 |
| GAPDH | Proteintech | 60004-1-Ig |
| HRP-Goat Anti-Rabbit IgG (H+L) | Proteintech | RGAR001 |
| HRP-Goat Anti-Mouse IgG (H+L) | Proteintech | SA00001-1 |

Supplementary Figure 1: Classification diagram of compound constituents in CAOL identified by UPLC-Q-TOF/MS.

Supplementary Figure 2: Schematic diagram of the experimental design.

Supplementary Figure 3: Representative M-mode echocardiographic images with anatomical labeling. M-mode tracings were obtained at the papillary muscle level in the parasternal long-axis view. IVS, interventricular septum; LVIDd, left ventricular internal diameter at end-diastole; LVIDs, left ventricular internal diameter at end-systole; LVPW, left ventricular posterior wall.

Supplementary Figure 4: A. Representative whole-heart Masson trichrome staining images for each group. Cardiac sections were stained with Masson trichrome to visualize collagen deposition (blue) and viable myocardium (red). Infarcted areas appear as blue-stained fibrotic tissue, predominantly localized in the left ventricular anterior wall. Scale bars = 2.5 mm. B. Representative H&E-stained images of cardiomyocyte morphology. High-magnification views of left ventricular sections from each group (scale bar = 50 μm). Cardiomyocytes in the model group exhibited enlarged cross-sectional areas compared with the sham group, while CAOL treatment dose-dependently attenuated these morphological changes.

Supplementary Figure 5: Serum biochemical parameters of liver and kidney function in each group. Data are presented as mean ± SD. ^#^P < 0.05, ^##^P < 0.01 compared with the sham group; *P < 0.05 compared with the model group; ns, non-significant. ALT, alanine aminotransferase; AST, aspartate aminotransferase; CREA, creatinine; UREA, urea nitrogen.
